# Application of propensity score matching in prognostic analysis of portal hypertension in hepatocellular carcinoma patients

**DOI:** 10.3389/fonc.2025.1644629

**Published:** 2026-01-12

**Authors:** Qiang Gao, Chunyi Zhu, Meifeng Chen, Shutian Mo, Yongfei He, Ketuan Huang, Yuan Liao, Tianyi Liang, Chuangye Han, Tao Peng

**Affiliations:** 1 Hepatobiliary Surgery, the Affiliated Hospital of Inner Mongolia Medical University, Inner Mongolia, China; 2 Hepatobiliary Surgery, the First Affiliated Hospital of Guangxi Medical University, Nanning, China; 3 Key Laboratory of Early Prevention and Treatment for Regional High Frequency Tumor (Guangxi Medical University), Ministry of Education, Nanning, China

**Keywords:** hepatocellular carcinoma (HCC), portal hypertension (PHT), prognostic analysis, propensity score matching (PSM), splenic modulation procedures

## Abstract

**Objective:**

To investigate the impact of portal hypertension on surgical prognosis after hepatectomy for hepatocellular carcinoma and assess the therapeutic value of concomitant splenic modulation procedures.

**Methods:**

We retrospectively analyzed HCC patients who underwent open hepatectomy with intraoperative portal venous pressure (PVP) measurement at our center between January 2013 and January 2020. Portal hypertension (PHT) was defined as PVP ≥ 25 cm H_2_O. Patients were categorized as PHT (n = 88) or non-PHT (n = 642). Propensity score matching (1:1) was performed to balance baseline covariates; matched analyses included 59 pairs. Short-term perioperative outcomes and long-term overall survival (OS) and recurrence-free survival (RFS) were compared between groups. Within the PHT cohort, outcomes were compared between hepatectomy alone and hepatectomy combined with splenectomy or splenic artery ligation.

**Results:**

Post-PSM analysis (59 matched pairs) revealed worse short-term outcomes in the PHT group: shorter surgical duration (p < 0.05) but greater intraoperative blood loss (anatomical/extensive hepatectomy subgroups, p < 0.01), higher postoperative bilirubin levels (p < 0.05), and longer hospital stay (non-anatomical/non-extensive hepatectomy subgroups, p < 0.01). Long-term OS and RFS showed no differences between groups pre- or post-PSM (p > 0.05). However, subgroup analyses demonstrated superior RFS in non-PHT patients undergoing non-anatomical and non-extensive hepatectomy (p = 0.035/0.034). Notably, pre-PSM data indicated improved RFS and OS in PHT patients receiving concomitant splenectomy or splenic artery ligation versus hepatectomy alone (p < 0.001).

**Conclusion:**

Compared with non-PHT patients, PHT was not associated with additional risk factors for poor prognosis after surgery. However, PHT may represent a significant risk indicator for recurrence in HCC patients undergoing non-anatomical or non-extensive hepatectomy. Furthermore, for HCC patients with concomitant PHT, hepatectomy combined with splenic artery ligation or splenectomy was associated with better long-term survival.

## Background

Hepatocellular carcinoma (HCC), the fourth leading cause of cancer-related mortality globally, has a particularly high incidence in cirrhotic populations (1, 2). In China, 86% of HCC cases arise in cirrhotic livers (3), and 31.2% present with concurrent portal hypertension (PHT) (4), a critical comorbidity that influences therapeutic decision-making.

Early evidence established PHT severity as an independent predictor of postoperative hepatic failure following hepatectomy (5, 6). Meta-analyses further confirm that clinically significant portal hypertension (CSPH) negatively impacts both surgical mortality and 5-year survival rates in HCC patients undergoing hepatectomy (4, 7). These risks underpin international guidelines from the Barcelona Clinic Liver Cancer (BCLC) and the European Association for the Study of the Liver (EASL), which contraindicate hepatectomy in patients with PHT (8, 9). However, the China Liver Cancer (CNLC) staging system permits surgical intervention for selected resectable HCC cases with PHT (10), creating therapeutic ambiguity that requires resolution through robust clinical evidence.

While hepatectomy remains the first-line therapy for early-stage HCC with preserved liver function (9), its application in patients with PHT remains contentious. PHT correlates with increased postoperative morbidity and risk of hepatic decompensation, traditionally rendering it a surgical contraindication (6, 11, 12). Recent EASL guidelines propose a risk-stratification algorithm incorporating PHT status, extent of hepatectomy, and the Model for End-Stage Liver Disease (MELD) score to guide hepatectomy eligibility (13). However, this framework is derived mainly from open-surgery cohorts using indirect PHT surrogates, whereas laparoscopic approaches have been shown to reduce hepatic injury (14–17). Crucially, the gold-standard hepatic venous pressure gradient (HVPG) measurement for CSPH diagnosis is underrepresented in Eastern populations, limiting the applicability of Western-derived algorithms to Chinese clinical practice (18, 19).

This study aimed to retrospectively analyze clinical data from patients with hepatocellular carcinoma undergoing hepatectomy using propensity score matching. By systematically comparing the clinical characteristics, perioperative outcomes, and long-term prognosis between the PHT and non-PHT groups, and further evaluating the efficacy of concomitant splenic modulation within the PHT cohort, this research sought to elucidate the impact of portal hypertension on surgical prognosis. It provides real-world evidence based on direct portal venous pressure measurements for an East Asian population, and offers a comprehensive assessment of the value of splenic modulation procedures, thereby addressing a significant gap in the existing literature.

## Materials and methods

### Study population

A total of 1,187 hepatocellular carcinoma (HCC) patients who underwent open hepatectomy with intraoperative portal pressure measurement at the First Affiliated Hospital of Guangxi Medical University between January 2013 and January 2020 were initially enrolled. The study protocol was approved by the Ethics Committee of the First Affiliated Hospital of Guangxi Medical University (Approval No. 2023-E488-01). The inclusion and exclusion criteria were as follows:

Inclusion criteria: (1) patients who underwent open hepatectomy with intraoperative portal pressure measurement at our hospital between January 2013 and January 2020; (2) age ≥ 18 and < 70 years; (3) postoperative histopathological confirmation of hepatocellular carcinoma.

Exclusion criteria: (1) patients with postoperative recurrence, ruptured HCC, previous upper abdominal surgery, any preoperative interventional therapy (such as TACE or HAIC), portal vein ligation, splenectomy, or splenic artery ligation; (2) presence of portal vein or splenic vein tumor thrombus, macrovascular invasion, or arteriovenous fistula; (3) lack of complete preoperative contrast-enhanced CT imaging; (4) missing preoperative or postoperative laboratory tests (including complete blood count, liver function, coagulation profile, etc.); (5) coexistence of other malignancies; (6) loss to follow-up after surgery.

**Figure 1:**
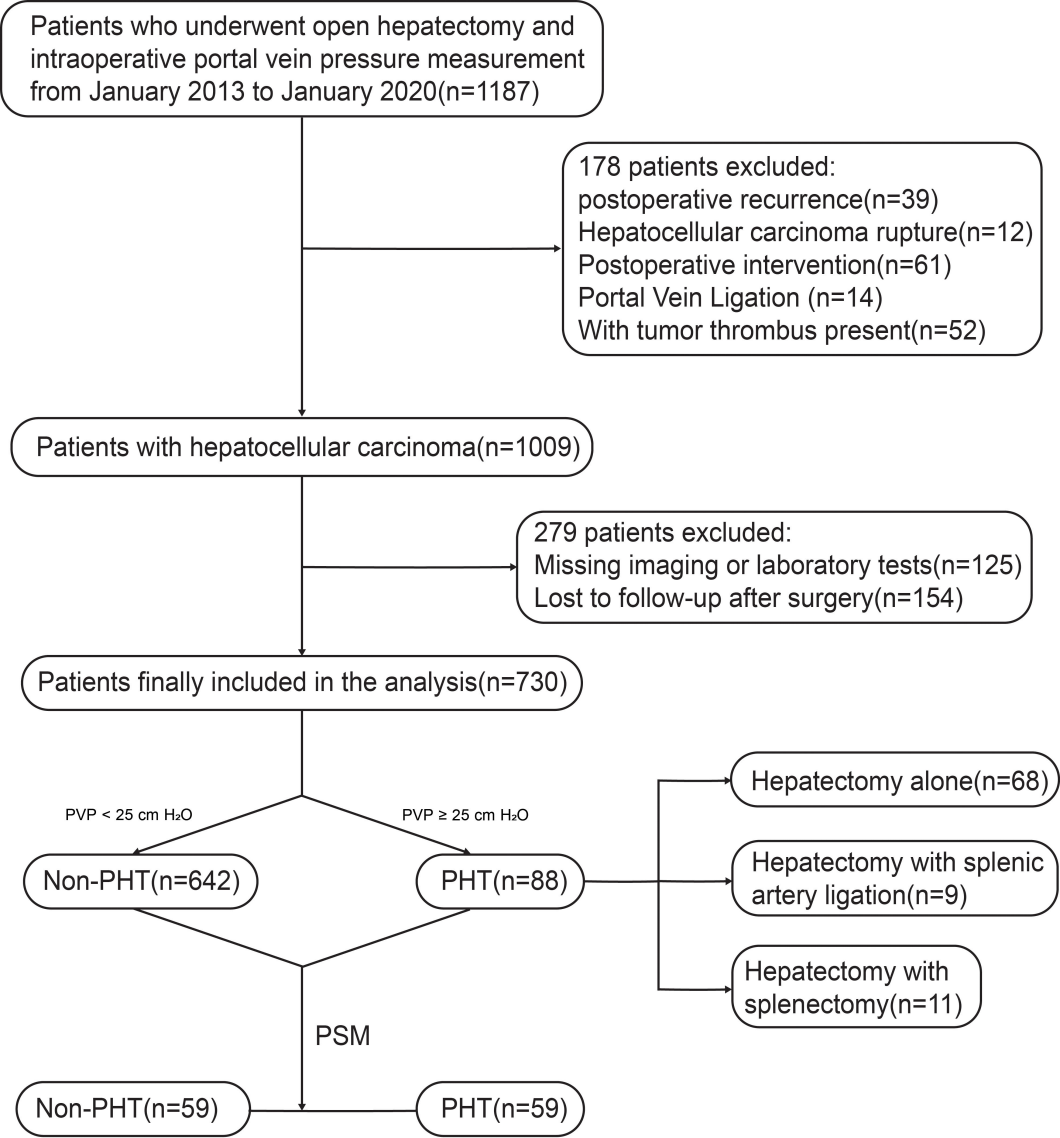
Flow chart for patient selection and propensity score matching process. The updated flowchart illustrates the initial patient screening, application of inclusion/exclusion criteria resulting in the final cohort of 730 patients (PHT n=88, non-PHT n=642), and subsequent 1:1 propensity score matching yielding 59 pairs for analysis. The PHT group is further stratified to show patients who did or did not undergo concomitant splenic modulation procedures.

After applying the above criteria, a total of 730 HCC patients were ultimately included in the study. (**Figure 1**)

### Data collection

Clinical and imaging parameters were extracted from the institutional electronic medical records system of the First Affiliated Hospital of Guangxi Medical University. The collected data included: patient demographics; etiology of liver disease; serological testing for liver fluke infection performed using an enzyme-linked immunosorbent assay to detect serum IgG antibodies against *Clonorchis sinensis*; laboratory parameters (e.g., platelet count, liver function tests, INR), from which APRI and FIB-4 scores and Child–Pugh grade were derived; preoperative imaging metrics measured from CT scans, including portal vein diameter (PVD), splenic vein diameter (SVD), liver volume (LV), and spleen volume (SV); surgical details including anatomical vs. non-anatomical and extensive vs. non-extensive hepatectomy, intraoperative blood loss, operation time, and concurrent procedures (splenectomy or splenic artery ligation); the core study variable of intraoperative portal venous pressure (PVP); tumor characteristics (e.g., size, number, greatest tumor diameter [GTD], microvascular invasion [MVI], AFP levels, abnormal prothrombin levels (also known as des-gamma-carboxy prothrombin, DCP), BCLC and CNLC stage); and outcome measures covering short-term endpoints (e.g., postoperative bilirubin, hospital stay, Clavien–Dindo complication grade) and long-term survival (overall survival [OS] and recurrence-free survival [RFS]).

### Follow-up

Patients were regularly followed up through outpatient clinic visits, imaging reviews, and telephone interviews until the cutoff date of December 31, 2023. In this study, recurrence-free survival (RFS) was defined as the time from the date of surgery to the date of recorded tumor recurrence or the last follow-up. Overall survival (OS) was defined as the time from the date of surgery to the date of patient death or the last follow-up. The median follow up duration for the entire cohort was 52.3 months (IQR 29.5–76.1). For the PHT group, the median follow up was 49.8 months (IQR 28.3–74.2), and for the non PHT group, it was 52.8 months (IQR 29.8–76.4).

### CT imaging - related Indicators

Preoperative contrast-enhanced CT scans were analyzed using 3D-Slicer (www.slicer.org) with the Total Segmentation plugin to calculate: portal vein diameter (PVD) at the confluence of the left and right branches; splenic vein diameter (SVD) at its proximal origin (two independent measurements averaged for final values); total liver volume (LV); and spleen volume (SV). Two board-certified radiologists independently performed manual ROI segmentation using ITK-SNAP (http://www.itksnap.org), and tumor-to-liver volume ratios were calculated for functional liver remnant assessment.

### Portal pressure measurement

Intraoperative portal venous pressure (PVP) was measured under general anesthesia via right gastroepiploic vein cannulation using a saline-filled manometer (zero reference: mid-axillary line). PHT was defined as PVP ≥ 25 cm H_2_O (20).

Indications and criteria for splenic modulation procedures: For HCC patients complicated by portal hypertension (PHT), the decision to perform a concomitant splenic modulation procedure (splenectomy or splenic artery ligation) during hepatectomy was made according to the following criteria.

Indications for Splenic Modulation: The procedure was indicated only when all the following conditions were met: (1) Adequate hepatic function (Child-Pugh class A or strictly selected class B with a score ≤7); (2) Significantly elevated portal venous pressure (intraoperative measurement ≥35 cm H_2_O); (3) Presence of marked hypersplenism, particularly when the platelet count was < 80 × 10^9^/L.

Selection of Surgical Technique: The specific technique was chosen on an individualized basis. Splenectomy was performed for patients with intraoperative findings or preoperative evidence of moderate-to-severe esophagogastric varices or massive splenomegaly, provided that the peri-splenic anatomy was clear and the procedure was not anticipated to significantly increase surgical risk or duration. Splenic artery ligation served as an alternative, suitable for cases with milder varices, or when the planned hepatectomy itself was highly extensive, time-consuming, and the patient’s condition did not permit further prolongation or expansion of the surgery.

Contraindications: The presence of Child-Pugh-C cirrhosis, uncontrolled active infection, or severe coagulopathy was considered an absolute contraindication for splenic modulation.

### Statistical methods

Propensity score matching (1:1) was used to balance 18 baseline covariates between the PHT and non-PHT cohorts, including demographic (sex), biochemical (platelet count, total bilirubin, albumin, AST, ALT, AST/ALT ratio, APRI, INR, FIB-4), clinical (Child–Pugh score, presence of ascites), and imaging metrics (spleen volume, tumor/non-tumor liver volume ratio, portal and splenic vein diameters, maximal tumor diameter). To this end, the propensity score was calculated by enrolling these 18 variables into a logistic regression equation. Subsequently, patients in the non-PHT and PHT groups were matched 1:1 based on their raw propensity score without replacement, using the greedy matching method with a fixed caliper width of 0.5 standard deviation.

Continuous variables were presented as mean ± standard deviation for normally distributed data and compared using Student’s t test, or as median (interquartile range [IQR]) and compared using the Mann–Whitney U test for non-normally distributed data. Categorical variables were expressed as counts (percentages) and compared using Pearson’s chi-square test or Fisher’s exact test, as appropriate. Survival outcomes were analyzed using the Kaplan–Meier method and compared with the log-rank test. Missing data (< 5% of variables) were handled by mean imputation. Statistical significance was defined as p < 0.05.

## Results

### Baseline data of the study population

The study cohort comprised 730 patients underwent open hepatectomy for resectable hepatocellular carcinoma (HCC), stratified by intraoperative portal venous pressure measurements into portal hypertension (PHT, n = 88, 12%) and non-PHT (n = 642, 88%) groups. Significant intergroup disparities (p < 0.05) were observed across 17 parameters: demographic (gender), biochemical (platelet count, total bilirubin, albumin, AST, AST/ALT ratio, APRI, INR, FIB-4), clinical (Child-Pugh score, ascites), and imaging metrics (spleen volume, tumor/non-tumor liver volume ratios, portal/splenic vein diameters, maximal tumor diameter, CNLC stage) (**Table 1**).

**Table 1:** Comparison of the base line parameters between PHT and non-PHT group pre-PSM.

Characteristic	Non - PHT (N = 642)	PHT (N = 88)	P - value
Gender			0.027
Female	85 (13.2%)	20 (22.7%)	
Male	557 (86.8%)	68 (77.3%)	
Age, years	49.7 ± 11.2	51.5 ± 10.3	0.121
BMI, kg/m^2^	22.6 ± 3.17	23.2 ± 3.79	0.143
Diabetes			0.518
No	622 (96.9%)	84 (95.5%)	
Yes	20 (3.12%)	4 (4.55%)	
NLR	1.96 [1.45; 2.68]	2.04 [1.55; 2.81]	0.237
Plt, 10^9^/L	193 [149; 246]	108 [64.9; 169]	<0.001
Tbil, μmol/L	11.2 [8.20; 15.0]	15.1 [10.6; 19.7]	<0.001
Alb, g/L	39.6 [36.9; 41.9]	37.7 [34.8; 40.0]	<0.001
Ascites			0.03
No	520 (81.0%)	62 (70.5%)	
Yes	122 (19.0%)	26 (29.5%)	
AST, U/L	35.0 [26.0; 49.8]	39.5 [31.0; 54.5]	0.018
ALT, U/L	33.0 [24.0; 47.0]	32.0 [24.8; 53.8]	0.952
AST/ALT	1.00 [0.80; 1.40]	1.20 [0.90; 1.40]	0.003
APRI Score			<0.001
≤2	596 (92.8%)	70 (79.5%)	
>2	46 (7.17%)	18 (20.5%)	
INR	0.98 [0.92; 1.03]	1.02 [0.95; 1.10]	<0.001
Child - Pugh Score			<0.001
Class A	591 (92.1%)	60 (68.2%)	
Class B	51 (7.94%)	28 (31.8%)	
FIB - 4 Score			<0.001
Grade 1: <1.45	429 (66.8%)	25 (28.4%)	
Grade 2: 1.45 - 3.25	176 (27.4%)	34 (38.6%)	
Grade 3: >3.25	37 (5.76%)	29 (33.0%)	
Hepatitis B			0.693
No	116 (18.1%)	18 (20.5%)	
Yes	526 (81.9%)	70 (79.5%)	
Hepatitis C			0.232
No	631 (98.3%)	85 (96.6%)	
Yes	11 (1.71%)	3 (3.41%)	
HBV – DNA	1840 [500; 120250]	783 [500; 250000]	0.734
AFP, ng/mL			0.158
<400	437 (68.1%)	67 (76.1%)	
≥400	205 (31.9%)	21 (23.9%)	
Abnormal Prothrombin, ng/mL	307 [54.0; 2466]	174 [40.8; 743]	0.152
Liver Fluke Enzyme Marker			0.314
Negative	495 (77.1%)	62 (70.5%)	
Weakly Positive	60 (9.35%)	9 (10.2%)	
Positive	87 (13.6%)	17 (19.3%)	
SV, mm^3^	189632 [137196; 250850]	304958 [180239; 608712]	<0.001
LV, mm^3^	1244250 [1083682; 1460708]	1218790 [1018122; 1398368]	0.069
Liver Tumor Volume, mm^3^	69361 [22038; 227937]	27845 [13207; 104997]	<0.001
Non - tumor Liver Volume, mm^3^	1109289 [970195; 1263326]	1103947 [953668; 1224254]	0.523
Liver Tumor Volume Percentage, %	5.66 [2.00; 17.6]	2.64 [1.14; 7.73]	<0.001
Non - tumor Liver Volume Percentage, %	94.3 [82.4; 98.0]	97.4 [92.3; 98.9]	<0.001
PVD, mm	13.9 [12.7; 15.3]	16.4 [15.2; 17.7]	<0.001
SVD, mm	10.0 [9.22; 10.9]	11.3 [10.5; 12.9]	<0.001
Invasion of Segment 1			1
No	621 (96.7%)	86 (97.7%)	
Yes	21 (3.27%)	2 (2.27%)	
Invasion of Segment 2			0.658
No	585 (91.1%)	82 (93.2%)	
Yes	57 (8.88%)	6 (6.82%)	
Invasion of Segment 3			0.969
No	586 (91.3%)	81 (92.0%)	
Yes	56 (8.72%)	7 (7.95%)	
Invasion of Segment 4			0.665
No	541 (84.3%)	72 (81.8%)	
Yes	101 (15.7%)	16 (18.2%)	
Invasion of Segment 5			0.093
No	404 (62.9%)	64 (72.7%)	
Yes	238 (37.1%)	24 (27.3%)	
Invasion of Segment 6			0.52
No	374 (58.3%)	55 (62.5%)	
Yes	268 (41.7%)	33 (37.5%)	
Invasion of Segment 7			0.604
No	421 (65.6%)	61 (69.3%)	
Yes	220 (34.3%)	27 (30.7%)	
Invasion of Segment 8			1
No	398 (62.0%)	55 (62.5%)	
Yes	244 (38.0%)	33 (37.5%)	
Number of Invaded Liver Segments			0.578
1	266 (41.4%)	45 (51.1%)	
2	250 (38.9%)	31 (35.2%)	
3	66 (10.3%)	7 (7.95%)	
4	57 (8.88%)	5 (5.68%)	
5	2 (0.31%)	0 (0.00%)	
6	1 (0.16%)	0 (0.00%)	
Number of Tumors			0.917
Single	555 (86.4%)	77 (87.5%)	
Multiple	87 (13.6%)	11 (12.5%)	
Maximum Tumor Diameter, cm	5.50 [3.50; 9.00]	4.50 [3.00; 6.00]	0.002
CNLC Stage			0.003
Ia	255 (39.7%)	53 (60.2%)	
Ib	310 (48.3%)	26 (29.5%)	
IIa	59 (9.19%)	5 (5.68%)	
IIb	5 (0.78%)	1 (1.14%)	
IIIa	11 (1.71%)	3 (3.41%)	
IIIb	2 (0.31%)	0 (0.00%)	
BCLC Stage			0.513
A	565 (88.0%)	78 (88.6%)	
B	64 (9.97%)	7 (7.95%)	
C	13 (2.02%)	3 (3.41%)	
Anatomical hepatectomy			0.194
No	306 (47.7%)	49 (55.7%)	
Yes	336 (52.3%)	39 (44.3%)	
Extent of hepatectomy			0.083
Non-Extensive: <3 Liver Segments	507 (79.0%)	77 (87.5%)	
Extensive: ≥3 Liver Segments	135 (21.0%)	11 (12.5%)	

Data are presented as mean ± standard deviation for normally distributed continuous variables, median [interquartile range] for non-normally distributed continuous variables, and count (percentage) for categorical variables.

Notably, the PHT cohort demonstrated smaller tumor volume, reduced maximal diameters, and higher CNLC stage Ia prevalence compared to non-PHT counterparts. To mitigate selection bias, propensity score matching (1:1) generated balanced cohorts (59 pairs) with comparable baseline characteristics (**Table 2**).

**Table 2:** Comparison of the base line parameters between PHT and non-PHT group post-PSM.

Characteristic	Non - PHT (N = 59)	PHT (N = 59)	P - value
Gender			1
Female	14 (23.7%)	14 (23.7%)	
Male	45 (76.3%)	45 (76.3%)	
Age, years	47.0 ± 11.5	52.6 ± 10.9	0.007
BMI, kg/m^2^	22.8 ± 3.71	23.2 ± 4.27	0.518
Diabetes			0.679
No	55 (93.2%)	57 (96.6%)	
Yes	4 (6.78%)	2 (3.39%)	
NLR	2.02 [1.67; 2.61]	2.05 [1.55; 2.90]	0.908
Plt, 10^9^/L	168 [111; 200]	120 [77.6; 203]	0.063
Tbil, μmol/L	14.3 [10.0; 18.2]	13.9 [10.4; 18.3]	0.87
Alb, g/L	38.9 [35.5; 40.8]	38.0 [35.4; 39.8]	0.254
Ascites			0.505
No	48 (81.4%)	44 (74.6%)	
Yes	11 (18.6%)	15 (25.4%)	
AST, U/L	33.0 [26.0; 54.0]	39.0 [31.0; 53.0]	0.211
ALT, U/L	30.0 [22.5; 53.0]	33.0 [24.5; 50.5]	0.878
AST/ALT	1.10 [0.90; 1.45]	1.20 [0.95; 1.40]	0.298
APRI Score			1
≤2	47 (79.7%)	47 (79.7%)	
>2	12 (20.3%)	12 (20.3%)	
INR	0.98 [0.94; 1.05]	1.00 [0.94; 1.06]	0.635
Child - Pugh Score			1
Class A	47 (79.7%)	46 (78.0%)	
Class B	12 (20.3%)	13 (22.0%)	
FIB - 4 Score			0.974
Grade 1: <1.45	20 (33.9%)	20 (33.9%)	
Grade 2: 1.45 - 3.25	24 (40.7%)	23 (39.0%)	
Grade 3: >3.25	15 (25.4%)	16 (27.1%)	
Hepatitis B			1
No	11 (18.6%)	11 (18.6%)	
Yes	48 (81.4%)	48 (81.4%)	
Hepatitis C			0.679
No	55 (93.2%)	57 (96.6%)	
Yes	4 (6.78%)	2 (3.39%)	
HBV – DNA	1000 [500; 19700]	704 [500; 384750]	0.745
AFP, ng/mL			1
<400	44 (74.6%)	44 (74.6%)	
≥400	15 (25.4%)	15 (25.4%)	
Abnormal Prothrombin, ng/mL	353 [58.5; 1358]	161 [38.2; 949]	0.482
Liver Fluke Enzyme Marker			0.442
Negative	47 (79.7%)	41 (69.5%)	
Weakly Positive	5 (8.47%)	8 (13.6%)	
Positive	7 (11.9%)	10 (16.9%)	
SV, mm^3^	278560 [183015; 408632]	246093 [162773; 434182]	0.677
LV, mm^3^	1153090 [1053715; 1387860]	1220770 [1049875; 1385700]	0.899
Liver Tumor Volume, mm^3^	42025 [10498; 175442]	32534 [15095; 118687]	0.989
Non - tumor Liver Volume, mm^3^	1067422 [997895; 1178128]	1109729 [955597; 1205843]	0.786
Liver Tumor Volume Percentage, %	4.25 [0.95; 13.5]	3.01 [1.28; 10.7]	0.899
Non - tumor Liver Volume Percentage, %	95.7 [86.5; 99.1]	97.0 [89.3; 98.7]	0.899
PVD, mm	15.7 [14.1; 18.0]	16.1 [14.6; 17.0]	0.998
SVD, mm	11.1 [10.2; 12.4]	11.1 [10.5; 12.0]	0.931
Invasion of Segment 1			0.619
No	56 (94.9%)	58 (98.3%)	
Yes	3 (5.08%)	1 (1.69%)	
Invasion of Segment 2			1
No	55 (93.2%)	55 (93.2%)	
Yes	4 (6.78%)	4 (6.78%)	
Invasion of Segment 3			0.717
No	56 (94.9%)	54 (91.5%)	
Yes	3 (5.08%)	5 (8.47%)	
Invasion of Segment 4			0.798
No	51 (86.4%)	49 (83.1%)	
Yes	8 (13.6%)	10 (16.9%)	
Invasion of Segment 5			1
No	43 (72.9%)	43 (72.9%)	
Yes	16 (27.1%)	16 (27.1%)	
Invasion of Segment 6			0.574
No	33 (55.9%)	37 (62.7%)	
Yes	26 (44.1%)	22 (37.3%)	
Invasion of Segment 7			1
No	39 (66.1%)	39 (66.1%)	
Yes	20 (33.9%)	20 (33.9%)	
Invasion of Segment 8			0.699
No	40 (67.8%)	37 (62.7%)	
Yes	19 (32.2%)	22 (37.3%)	
Number of Invaded Liver Segments			0.804
1	30 (50.8%)	30 (50.8%)	
2	22 (37.3%)	20 (33.9%)	
3	3 (5.08%)	6 (10.2%)	
4	4 (6.78%)	3 (5.08%)	
Tumor Number			1
Single	53 (89.8%)	52 (88.1%)	
Multiple	6 (10.2%)	7 (11.9%)	
Maximum Tumor Diameter, cm	4.50 [3.00; 7.50]	4.50 [3.50; 6.25]	0.794
CNLC Stage			0.671
Ia	29 (49.2%)	35 (59.3%)	
Ib	26 (44.1%)	19 (32.2%)	
IIa	3 (5.08%)	3 (5.08%)	
IIb	0 (0.00%)	1 (1.69%)	
IIIa	1 (1.69%)	1 (1.69%)	
BCLC Stage			0.857
A	55 (93.2%)	53 (89.8%)	
B	3 (5.08%)	5 (8.47%)	
C	1 (1.69%)	1 (1.69%)	
Anatomical hepatectomy			0.197
No	26 (44.1%)	34 (57.6%)	
Yes	33 (55.9%)	25 (42.4%)	
Extent of hepatectomy			0.616
Non-Extensive: <3 Liver Segments	48 (81.4%)	51 (86.4%)	
Extensive: ≥3 Liver Segments	11 (18.6%)	8 (13.6%)	

### Information on hypersplenism and varices in the PHT cohort

Within the PHT group (n=88), 34 patients (38.6%) had platelet counts <80×10^9^/L, indicative of laboratory evidence of hypersplenism. Massive splenomegaly was defined as a spleen volume >1000 cm³ on preoperative CT volumetry (21, 22), and was present in 19 patients (21.6%). Moderate-to-severe esophagogastric varices were defined as grade 2 or higher on preoperative endoscopy or as the presence of varices with red color signs on imaging/operative notes (23), and were documented in 27 patients (30.7%).

### Intraoperative and postoperative comparison

Pre-matching analyses revealed significant disparities in surgical parameters between cohorts. The portal hypertension (PHT) group demonstrated shorter operative durations (median 200 vs 244 minutes, p = 0.026) but greater intraoperative hemorrhagic burden (500 vs 350 mL, p < 0.001). Postoperatively, PHT patients exhibited prolonged hyperbilirubinemia (days 3–5 peak: 24.6 vs 17.7 μmol/L, p = 0.004) and extended hospitalization (12 vs 9 days, p < 0.001). Although Clavien-Dindo grade≥II complications occurred more frequently in the PHT cohort (15.9% vs 10.3%), this difference lacked statistical significance (**Table 3** and **Supplementary Table S1**).

**Table 3:** Comparison of intraoperative and postoperative outcomes between PHT and non-PHT groups before and after propensity score matching.

Characteristic	Non-PHT Pre-PSM (N = 642)	PHT Pre-PSM (N = 88)	P-value Pre-PSM	Non-PHT Post-PSM (N = 59)	PHT Post-PSM (N = 59)	P-value Post-PSM
Surgical Duration, min	244 [180;327]	200 [133;306]	0.026	240 [180;305]	185 [120;300]	0.02
Intraoperative Blood Loss, ml	350 [200;538]	500 [238;800]	0.001	300 [150;575]	500 [200;850]	0.025
Perioperative RBC Transfusion, U	3.00 [1.50;4.00]	3.00 [2.00;5.62]	0.082	3.25 [1.50;4.00]	3.00 [2.00;5.62]	0.638
Perioperative Plasma Transfusion, ml	600 [400;600]	600 [400;600]	0.594	600 [450;600]	600 [400;600]	0.782
Presence of MVI			0.947			0.325
No	424 (66.0%)	59 (67.0%)		37 (62.7%)	43 (72.9%)	
Yes	218 (34.0%)	29 (33.0%)		22 (37.3%)	16 (27.1%)	
Bilirubin (3–5 days postoperative), μmol/L	17.7 [12.9;25.4]	24.6 [14.1;33.8]	0.004	18.6 [14.1;26.1]	24.0 [13.8;30.6]	0.554
Postoperative Hospital Stay, day	9.00 [8.00;12.0]	12.0 [9.75;18.0]	<0.001	10.0 [7.50;11.5]	11.0 [9.00;15.0]	0.004
Clavien-Dindo Grade			0.161			0.741
<3	576 (89.7%)	74 (84.1%)		55 (93.2%)	53 (89.8%)	
≥3	66 (10.3%)	14 (15.9%)		4 (6.78%)	6 (10.2%)	

### Intraoperative and postoperative comparison after PSM

Propensity score-matched cohorts (n = 59 per group) demonstrated persistent surgical disparities, with the portal hypertension (PHT) group maintaining shorter operative durations (median 185 vs 240 min, p = 0.020) and greater intraoperative blood loss (500 vs 300 mL, p = 0.025) compared to non-PHT counterparts. Notably, postoperative hyperbilirubinemia (days 3–5: 24.0 vs 18.6 μmol/L) lost statistical significance post-matching (p = 0.554). However, prolonged hospitalization remained evident in PHT patients (11 vs 10 days, p = 0.004). No significant intergroup differences were observed in transfusion requirements (RBC: 3.00 vs 3.25 U, p = 0.638; FFP: 600 vs 600 mL, p = 0.782), microvascular invasion prevalence (27.1% vs 37.3%, p = 0.325), or Clavien-Dindo grade ≥III complications (10.2% vs 6.78%, p = 0.741) (**Table 3**).

### Subgroup analysis

In the propensity score-matched cohort undergoing Anatomical hepatectomy (PHT: n = 25 vs non-PHT: n = 33), the portal hypertension group demonstrated significantly higher intraoperative blood loss (median 500 vs 250 mL, p = 0.015) and a clinically notable increase in Clavien-Dindo grade ≥III complications (20.0% vs 3.03%, p = 0.075), although overall complication rates remained comparable (p = 0.882). No intergroup differences were observed in operative duration (243 vs 267 min, p = 0.377), transfusion requirements (RBC: 2.75 vs 4.00 U, p = 0.960; FFP: 550 vs 500 mL, p = 0.650), microvascular invasion prevalence (28.0% vs 33.3%, p = 0.882), postoperative hyperbilirubinemia (25.2 vs 18.6 μmol/L, p = 0.718), or hospitalization length (10 vs 10 days, p = 0.554).

Patients undergoing non-Anatomical hepatectomy with portal hypertension (PHT, n = 34) demonstrated significantly prolonged postoperative hospitalization compared to non-PHT counterparts (median 12 [IQR 10.2-15] vs 10 [7-11] days, p < 0.001), despite comparable intraoperative metrics including blood loss (400 vs 350 mL, p = 0.508) and operative duration (180 vs 225 min, p = 0.070). No significant intergroup differences were observed in transfusion requirements (RBC: 3.00 vs 2.25 U; FFP: 600 vs 600 mL), microvascular invasion rates (26.5% vs 42.3%), or Clavien-Dindo grade ≥III complications (2.94% vs 11.5%).

In patients undergoing extensive hepatectomy, the portal hypertension (PHT) cohort exhibited significantly greater intraoperative blood loss compared to non-PHT counterparts (median 900 vs 200 mL, p = 0.011), despite comparable surgical durations (330 vs 253 min, p = 0.094) and transfusion requirements (RBC: 2.75 vs 4.00 U, p = 0.207; FFP: 600 vs 500 mL, p = 0.801). No intergroup differences were observed in postoperative outcomes, including hyperbilirubinemia (26.5 vs 27.1 μmol/L, p = 0.602), hospitalization length (9.5 vs 10 days, p = 0.403), or severe complications (Clavien-Dindo ≥III: 25.0% vs 0%, p = 0.164).

Patients undergoing non-extensive hepatectomy with portal hypertension (PHT, n = 51) demonstrated reduced operative durations (median 180 vs 240 min, p = 0.004) but prolonged postoperative hospitalization (11 vs 10 days, p = 0.001) compared to non-PHT counterparts (n = 48). No significant intergroup disparities were observed in intraoperative blood loss (400 vs 300 mL, p = 0.240), transfusion volumes (RBC: 3.00 vs 2.00 U; FFP: 600 vs 600 mL), microvascular invasion rates (27.5% vs 39.6%), or Clavien-Dindo ≥III complications (7.84% vs 8.33%). Postoperative hyperbilirubinemia showed comparable trajectories (21.6 vs 18.1 μmol/L, p = 0.435) (**Table 4**).

**Table 4:** Comparative Surgical Outcomes Between PHT and non-PHT Groups Across hepatectomy Types After PSM.

Characteristic	Anatomical hepatectomy	Non-anatomical hepatectomy	Large-scale hepatectomy	Non-extensive hepatectomy
Sample Size	Non-PHT:33 PHT:25	Non-PHT:26 PHT:34	Non-PHT:11 PHT:8	Non-PHT:48 PHT:51
Surgical Duration, min				
Non-PHT	267 [189-311]	225 [158-268]	253 [196-310]	240 [180-304]
PHT	243 [165-318]	180 [120-267]	330 [275-360]	180 [120-267]
p-value	0.377	0.07	0.094	0.004
Intraoperative Blood Loss, ml				
Non-PHT	250 [150-500]	350 [200-800]	200 [100-475]	300 [180-550]
PHT	500 [200-900]	400 [212-800]	900 [575-1200]	400 [200-800]
p-value	0.015	0.508	0.011	0.24
Perioperative RBC Transfusion, U				
Non-PHT	4.00 [2.50-4.00]	2.25 [1.50-4.25]	4.00 [4.00-6.25]	2.00 [1.50-3.75]
PHT	2.75 [2.25-5.50]	3.00 [1.50-5.50]	2.75 [2.50-4.00]	3.00 [1.50-6.00]
p-value	0.96	0.55	0.207	0.345
Perioperative Plasma Transfusion, ml				
Non-PHT	500 [400-600]	600 [600-600]	500 [450-550]	600 [550-600]
PHT	550 [400-600]	600 [512-600]	600 [400-600]	600 [400-600]
p-value	0.65	0.6	0.801	0.624
MVI Positivity				
Non-PHT	11 (33.3%)	11 (42.3%)	3 (27.3%)	19 (39.6%)
PHT	7 (28.0%)	9 (26.5%)	2 (25.0%)	14 (27.5%)
p-value	0.882	0.311	1	0.286
Bilirubin (3–5 days postoperative), μmol/L				
Non-PHT	18.6 [12.2-30.4]	18.4 [15.9-22.9]	27.1 [16.7-45.7]	18.1 [14.1-24.0]
PHT	25.2 [13.8-27.9]	21.8 [14.0-31.3]	26.5 [24.0-27.6]	21.6 [13.8-31.0]
p-value	0.718	0.8	0.602	0.435
Postoperative Hospital Stay, day				
Non-PHT	10.0 [8.0-12.0]	10.0 [7.0-11.0]	10.0 [10.0-12.0]	10.0 [7.0-11.0]
PHT	10.0 [9.0-14.0]	12.0 [10.2-15.0]	9.5 [8.8-11.8]	11.0 [9.5-15.5]
p-value	0.554	0.001	0.403	0.001
Clavien-Dindo Grade (≥Grade 3)				
Non-PHT	1 (3.03%)	3 (11.5%)	0 (0.00%)	4 (8.33%)
PHT	5 (20.0%)	1 (2.94%)	2 (25.0%)	4 (7.84%)
p-value	0.075	0.307	0.164	1

### Long - term prognosis comparison

**Figure 2:**
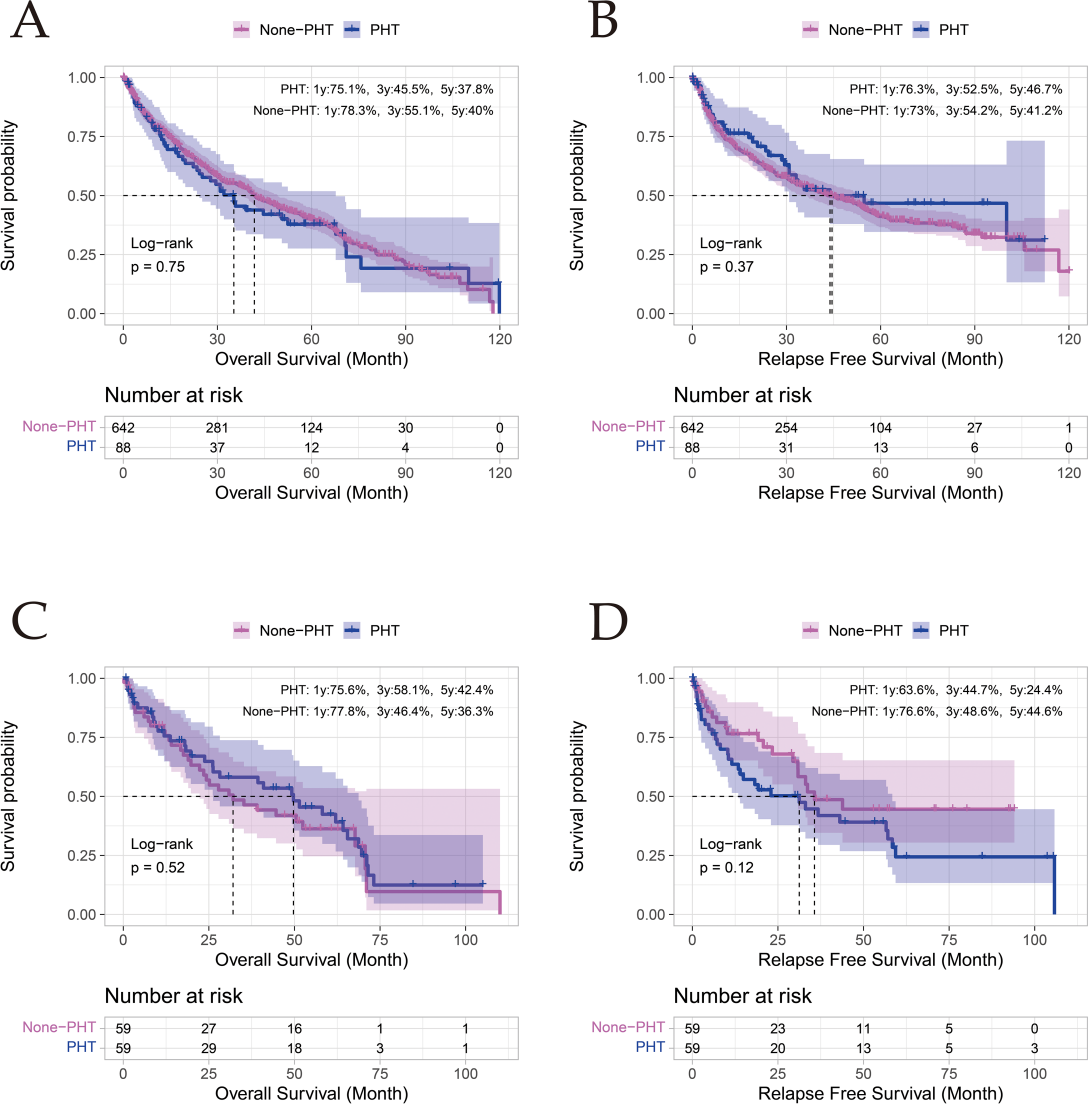
Comparison of OS and RFS between the PHT and non - PHT Groups Before and After PSM. **(A, B)** represent OS and RFS before propensity score matching (PSM), respectively; Panels **(C, D)** represent OS and RFS after PSM, respectively.

Propensity score-matched analyses demonstrated comparable long-term survival outcomes between PHT and non-PHT cohorts across all evaluated timepoints. Pre-matching 5-year overall survival (OS) rates were 37.8% (PHT) versus 40% (non-PHT) (p = 0.750), with recurrence-free survival (RFS) at 46.7% versus 41.2% (p = 0.370). Post-matching analyses revealed persistent non-significant disparities: 5-year OS of 42.4% (PHT) versus 36.3% (non-PHT) (p = 0.520), and RFS of 24.4% versus 44.6% (p = 0.120). Notably, the 3-year OS paradoxically favored PHT patients post-PSM (58.1% vs 46.4%), though this difference remained statistically insignificant. These findings collectively indicate that portal hypertension status does not independently predict long-term survival outcomes following hepatectomy (**Figure 2**; **Supplementary Table S2**).

### Subgroup analysis of long - term prognosis

**Figure 3:**
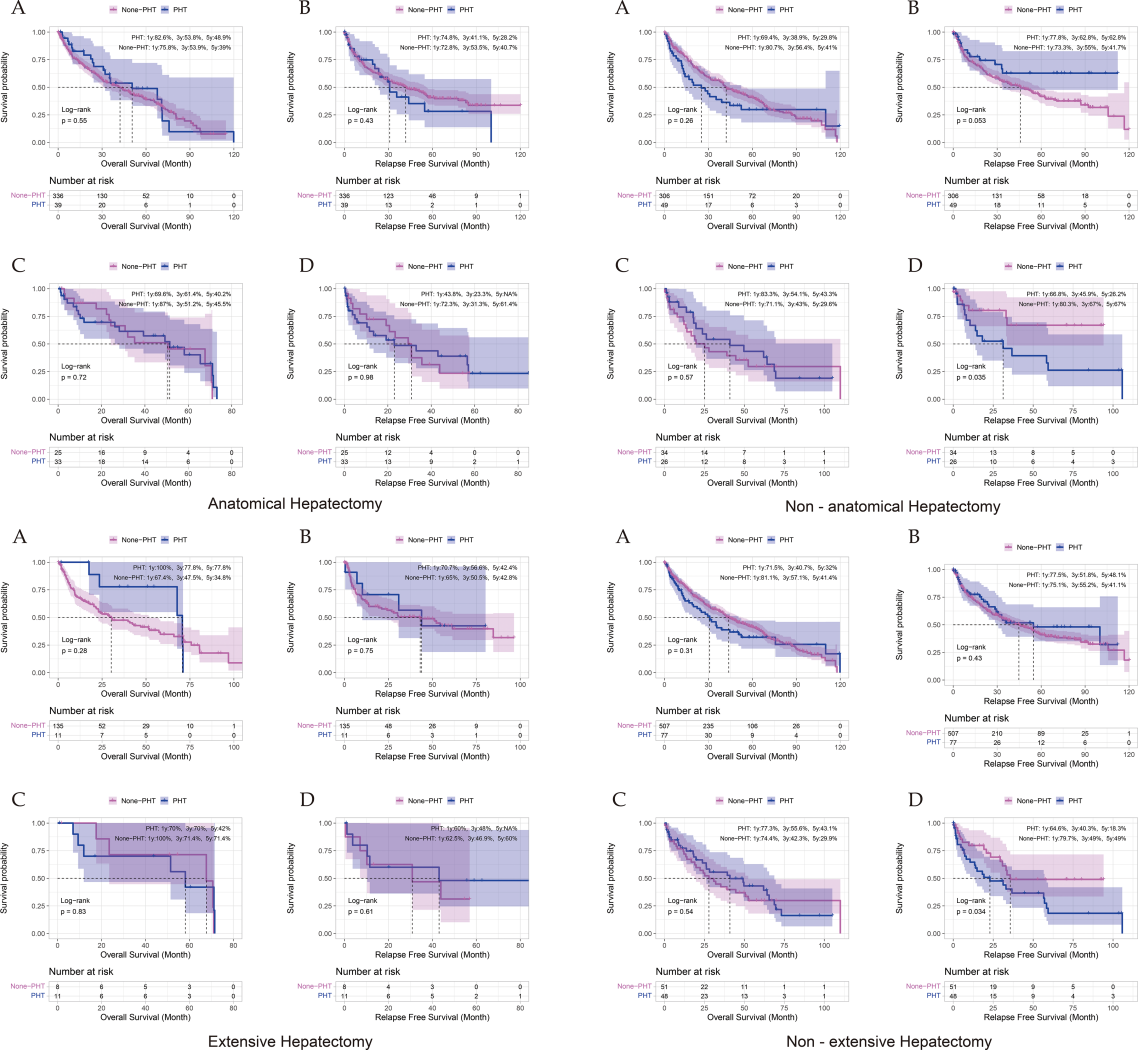
Comparison of Survival Outcomes by Surgical Type and PSM Stage. **(A, B)** represent OS and RFS before propensity score matching (PSM), respectively; Panels **(C, D)** represent OS and RFS after PSM, respectively.

Subgroup analyses revealed distinct survival patterns based on hepatectomy types. For anatomical hepatectomies, comparable 5-year overall survival (OS) and recurrence-free survival (RFS) were observed between portal hypertension (PHT) and non-PHT cohorts both pre- (OS: 48.9% vs 39%, p = 0.550; RFS: 28.2% vs 40.7%, p = 0.430) and post-propensity score matching (PSM) (OS: 40.2% vs 45.5%, p = 0.720). Conversely, non-anatomical hepatectomy demonstrated significant RFS disparities post-PSM (26.2% vs 67%, p = 0.035). Extensive hepatectomies showed no survival differences across all stages (5-year OS post-PSM: 42% vs 71.4%, p = 0.830). Notably, non-extensive hepatectomy exhibited significantly inferior RFS in PHT patients post-PSM (18.3% vs 49%, p = 0.030, suggesting residual portal hypertension pathophysiology adversely impacts recurrence in parenchymal-sparing procedures (**Figure 3**; **Supplementary Table S3**).

### Long - term prognosis of the PHT group

**Figure 4:**
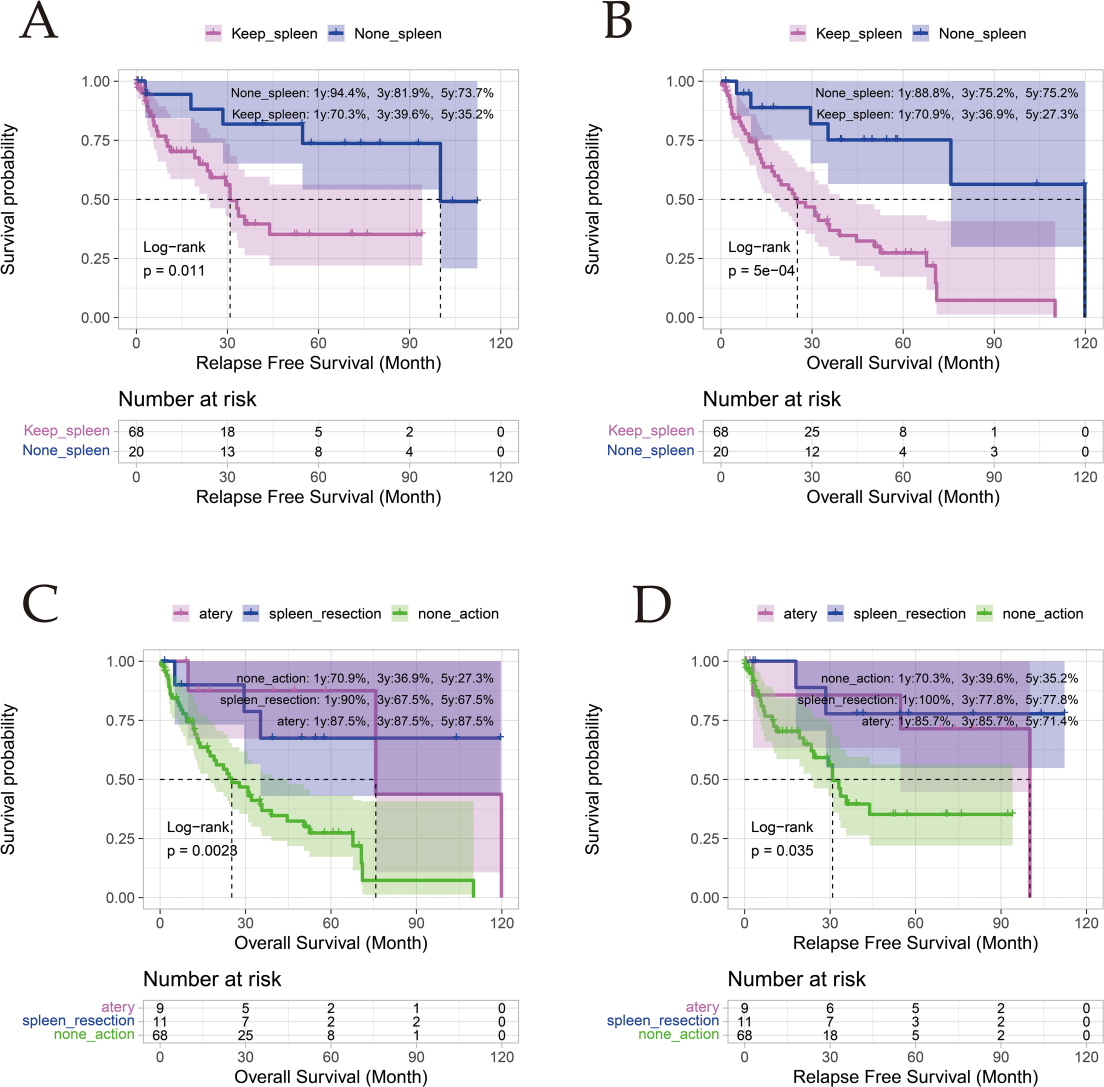
Comparison of Survival Outcomes Among Different Spleen Interventions in the PHT Group. **(A, B)** depict Relapse-Free Survival (RFS) and Overall Survival (OS) for the splenectomy group and the conservative management group, respectively; Panels **(C, D)** depict OS and RFS for the splenectomy group, splenic artery ligation group, and conservative management group, respectively.

In patients with portal hypertension (PHT) undergoing open hepatectomy, three spleen management strategies were analyzed: hepatectomy alone (n = 68), hepatectomy with splenectomy (n = 11), and hepatectomy with splenic artery ligation (n = 9). Given limited sample sizes precluding propensity score matching, direct comparisons revealed no survival differences between splenectomy and splenic artery ligation cohorts (5-year OS: 67.0% vs 87.5%, p = 0.800; RFS: 77.8% vs 71.4%, p = 0.440). However, both intervention groups demonstrated superior outcomes compared to hepatectomy-alone controls, with splenectomy achieving 67.0% vs 27.3% 5-year OS (p = 0.0028) and 77.8% vs 35.2% RFS (p = 0.035), while splenic artery ligation showed 87.5% vs 27.3% OS (p = 0.0028) and 71.4% vs 35.2% RFS (p = 0.035). These findings suggest adjunctive splenic modulation may mitigate PHT-related survival deficits, though larger cohorts are needed for validation (**Figure 4**; **Supplementary Tables S4**, **S5**).

## Discussion

Hepatectomy remains the primary curative intervention for hepatocellular carcinoma (HCC) in patients with preserved liver function (24). However, no universally established guidelines exist for managing HCC with concurrent portal hypertension (PHT). The 2022 updated Barcelona Clinic Liver Cancer (BCLC) staging system explicitly contraindicates hepatectomy in patients with elevated portal venous pressure due to concerns over postoperative hepatic decompensation (25), and instead recommends liver transplantation as a key treatment option for BCLC-A patients with solitary nodules and clinically significant portal hypertension (8). The patients in this study underwent hepatectomy based on real-world clinical decisions, which may be influenced by factors such as organ availability, patient preference, or transplant contraindications. As a retrospective analysis, our data cannot retrospectively ascertain the specific reasons for not pursuing transplantation in these individual cases. Therefore, the aim of this study was not to challenge the transplant recommendation, but to evaluate the oncological outcomes of hepatectomy in a selected cohort of PHT patients who actually received this treatment, providing evidence for scenarios where transplantation is not feasible. Early studies identified PHT as an independent predictor of postoperative liver failure and reduced overall survival (OS) following hepatectomy (6, 26). Contrasting this historical perspective, recent multicenter studies demonstrate comparable postoperative complication rates and long-term survival between PHT and non-PHT cohorts (27–29). Cucchetti et al. conducted propensity-matched analyses of 89 PHT and 152 non-PHT patients, revealing statistically equivalent 5-year OS rates (56.3% vs 61.4%, p = 0.380) (30).Chinese clinical series further corroborate these findings, with multiple studies reporting preserved survival outcomes in carefully selected PHT patients (27, 28). Consistent with prior evidence, our propensity score-matched analysis revealed comparable baseline characteristics and survival outcomes between portal hypertension (PHT) and non-PHT cohorts. Both pre- and post-matching comparisons demonstrated non-significant disparities in OS and RFS, indicating that rigorously selected PHT patients undergoing standardized perioperative protocols do not incur additional prognostic risks compared to non-PHT counterparts. These findings support surgical intervention as a viable option for compensated PHT-HCC patients. Notably, comparable microvascular invasion (MVI) rates were observed, likely attributable to the predominance of early-stage tumors in our PHT cohort. Smaller tumor volumes at this disease stage may limit vascular infiltration capacity, potentially explaining the preserved oncological outcomes.

The literature on perioperative outcomes for patients with portal hypertension (PHT) is extensive but not entirely consistent. Several retrospective cohort studies and multicenter database analyses that used HVPG or clinical surrogates (such as splenomegaly, thrombocytopenia, or esophagogastric varices) have shown that patients with clinically significant portal hypertension (CSPH—commonly defined as HVPG ≥10 mmHg or by clinical surrogates) undergoing hepatectomy overall experience higher rates of postoperative hepatic decompensation, increased perioperative mortality, and worse long-term survival (31–33). However, some studies using propensity score matching (PSM) or multivariable adjustment indicate that, in carefully selected compensated patients, those with PHT who undergo hepatectomy may achieve long-term survival and complication rates comparable to non-PHT patients (34, 35). Consistent with these reports, our study also observed shorter surgical duration in the PHT group, largely because these patients had smaller tumor volumes, shorter maximal diameters, and higher proportions of early-stage disease—factors that reduced operative complexity. Nonetheless, both in our cohort and in many prior studies, PHT patients commonly face higher intraoperative blood loss, particularly during anatomical and extensive hepatectomy, which may relate to thrombocytopenia, increased portal/intrahepatic venous pressures, and development of portosystemic collaterals (36–39). To mitigate these risks, international guidelines and expert consensuses recommend multimodal hemorrhage control and liver-protection measures—our study followed the 2020 expert consensus, implementing ultrasound-guided hepatectomy planning, the Pringle maneuver with selective inflow occlusion, intraoperative low CVP management (<5 mmHg), and coagulation factor replacement (9, 40, 41). Despite these measures, PHT patients undergoing non-anatomical or non-extensive hepatectomy still showed prolonged postoperative hospitalization, and Clavien–Dindo ≥III complications were numerically higher than in non-PHT patients though not statistically significant. This observation is concordant with some laparoscopic series (42), suggesting that modern minimally invasive and precision surgical techniques can partially mitigate classical PHT-associated risks. However, current evidence is limited by retrospective designs and sample sizes; larger multicenter prospective studies are still required to validate and refine PHT-specific perioperative strategies.

Multiple studies have suggested that PHT is an independent risk factor for HCC recurrence after hepatectomy, particularly in patients who undergo non-anatomical or non-extensive hepatectomy (43–45). Specifically, several retrospective cohort and multicenter studies using clinical or hemodynamic criteria to define PHT found an association between PHT and increased intrahepatic recurrence after multivariable adjustment; this effect appears especially pronounced when conservative margins leave potential multifocal disease or micrometastases in situ (46–48). Our PSM subgroup analysis corroborated this: PHT patients who underwent non-anatomical or non-extensive hepatectomy had significantly worse recurrence-free survival (RFS) than matched non-PHT patients, while overall survival (OS) did not differ significantly, suggesting that PHT primarily impacts short- to mid-term outcomes by increasing intrahepatic recurrence. Mechanistically, basic and clinical research has proposed several pathways: PHT may induce sinusoidal architectural disruption, hepatic microcirculatory alterations, and local hypoxia, all of which can enhance residual tumor cell adaptability and activate EMT-related pathways (49, 50); concomitantly, PHT-associated impairment of liver regeneration and uneven distribution of drugs/metabolites may reduce the capacity to eradicate micrometastases postoperatively (51). In addition, the immune microenvironment in a PHT liver may be altered, reducing local immune surveillance and facilitating recurrence. Compared with studies that endorse non-extensive hepatectomy in carefully selected patients, our findings favor prioritizing anatomical hepatectomy or wider margins in compensated PHT patients whose liver function and remnant volume permit it, although this recommendation must be balanced against increased technical difficulty and the patient’s hepatic reserve and should be integrated with postoperative adjuvant or locoregional/systemic therapies to individualize care.

The value of concomitant splenic interventions (splenectomy or splenic artery ligation) combined with hepatectomy is supported by a body of retrospective evidence and several meta-analyses. Multiple comparative retrospective studies and meta-analyses have concluded that, in HCC patients with marked hypersplenism (e.g., substantial thrombocytopenia, massive splenomegaly) or elevated portal pressures, simultaneous splenectomy can significantly improve postoperative platelet and leukocyte counts, reduce perioperative bleeding issues, and in some reports is associated with better RFS and long-term survival; these studies generally stress that, when patients are carefully selected (compensated liver disease, adequate hepatic reserve, favorable splenic anatomy), the safety profile of concomitant splenectomy can be acceptable (34, 52–54). At the same time, splenic artery ligation has been proposed as a less invasive alternative that can partially reduce splenic inflow and portal pressure and produce short-term hematologic improvement, potentially conferring similar long-term benefits (43–45, 55); however, the evidence base for ligation is smaller and lacks large, long-term randomized head-to-head comparisons. In our study, despite limited numbers, both splenectomy and splenic artery ligation showed comparable survival outcomes and both appeared superior to hepatectomy alone, aligning with the directional findings of retrospective literature. It should be emphasized that while splenectomy may improve hemodynamics and possibly reduce recurrence risk, it substantially increases the incidence of portal venous thrombosis (PVT)—published series report a marked rise in PVT rates from the early postoperative period through the first year—so clinical decisions must carefully weigh individualized benefit against thrombotic and infectious risks (56). If splenectomy is performed, intensified postoperative PVT surveillance (early ultrasound/CT) and appropriate prophylactic/therapeutic anticoagulation should be considered. Overall, the available evidence supports considering splenic intervention under strict indications to improve outcomes in selected PHT patients, such as those with marked hypersplenism (particularly a platelet count < 80 × 10^9^/L), significantly elevated intraoperative portal venous pressure (e.g., ≥ 35 cm H_2_O), or concomitant moderate-to-severe esophagogastric varices or massive splenomegaly. However, definitive conclusions regarding its comparative efficacy, safety profile (especially the incidence of portal vein thrombosis), and impact on long-term survival require larger prospective or randomized trials.

This study has certain limitations that should be acknowledged. First, it focused exclusively on patients who underwent hepatectomy. Consequently, the findings primarily apply to this specific treatment cohort and may not be generalizable to all HCC patients with PHT, particularly those who are ideal candidates for and receive liver transplantation as per current guidelines. Second, the sample size, especially within the PHT subgroup undergoing different splenic procedures, was limited, which may affect the statistical power of the comparisons. Third, portal hypertension was defined by a single baseline intraoperative PVP measurement rather than the gold standard HVPG; although previous studies have demonstrated a strong correlation between PVP ≥ 25 cm H_2_O and HVPG ≥ 10 mmHg (57, 58), the absence of synchronous HVPG measurements in our cohort precluded direct validation of this agreement within our specific population. Dynamic postoperative PVP changes were not captured, potentially missing valuable hemodynamic information related to outcomes. Fourth, as a single-center retrospective analysis, the generalizability of our findings is constrained, and external validation from multi-center or prospective studies is necessary to confirm our conclusions.

## Conclusion

Our comparative analysis revealed distinct perioperative patterns between portal hypertension (PHT) and non-PHT cohorts. The PHT group exhibited significantly shorter operative durations but paradoxically greater intraoperative blood loss, subsequently requiring prolonged hospitalization. Notably, both groups maintained comparable 30-day complication rates. Long-term survival analysis demonstrated no significant intergroup differences in overall survival and recurrence-free survival. Importantly, subgroup analysis revealed that PHT patients undergoing limited hepatectomy (non-Anatomical/non-extensive hepatectomy) showed significantly inferior RFS compared to non-PHT counterparts, suggesting PHT’s role as a critical recurrence predictor in limited hepatectomy. Furthermore, combined surgical approaches integrating hepatectomy with splenic modulation (splenic artery ligation or splenectomy) demonstrated superior long-term survival outcomes in PHT patients, highlighting the potential benefits of portal flow modification strategies in this high-risk population.

## Data Availability

The original contributions presented in the study are included in the article/**Supplementary Material**. Further inquiries can be directed to the corresponding authors.

## Supplementary material

Supplementary Table 1 Clavien-Dindo grade (59).

Supplementary Table 2 Long - Term Prognosis Comparison.

Supplementary Table 3 Subgroup Analysis of Long - Term Prognosis.

Supplementary Table 4 Long - Term Prognosis of patients undergoing splenic modulation surgery.

Supplementary Table 5 Baseline characteristics sheet of patients undergoing splenic modulation surgery.

## References

[1] Villanueva A . Hepatocellular carcinoma. N Engl J Med. (2019) 380:1450–62. doi: 10.1056/NEJMra1713263, PMID: 30970190

[2] Alawyia B Constantinou C . Hepatocellular carcinoma: a narrative review on current knowledge and future prospects. Curr Treat Options Oncol. (2023) 24:711–24. doi: 10.1007/s11864-023-01098-9, PMID: 37103744

[3] Huang DQ Terrault NA Tacke F Gluud LL Arrese M Bugianesi E . Global epidemiology of cirrhosis - etiology, trends and predictions. Nat Rev Gastroenterol Hepatol. (2023) 20:388–98. doi: 10.1038/s41575-023-00759-2, PMID: 36977794 PMC10043867

[4] Liu J Zhang H Xia Y Yang T Gao Y Li J . Impact of clinically significant portal hypertension on outcomes after partial hepatectomy for hepatocellular carcinoma: a systematic review and meta-analysis. HPB (Oxford). (2019) 21:1–13. doi: 10.1016/j.hpb.2018.07.005, PMID: 30082213

[5] Vivarelli M Mocchegiani F Wakabayashi T Gaudenzi F Nicolini D Al-Omari MA . Prevention of post-hepatectomy liver failure in cirrhotic patients undergoing minimally invasive liver surgery for HCC: has the round ligament to be preserved? Cancers (Basel). (2024) 16(2):364. doi: 10.3390/cancers16020364, PMID: 38254855 PMC10814940

[6] Bruix J Castells A Bosch J Feu F Fuster J Garcia-Pagan JC . Surgical resection of hepatocellular carcinoma in cirrhotic patients: prognostic value of preoperative portal pressure. Gastroenterology. (1996) 111:1018–22. doi: 10.1016/s0016-5085(96)70070-7, PMID: 8831597

[7] Choi SB Kim HJ Song TJ Ahn HS Choi SY . Influence of clinically significant portal hypertension on surgical outcomes and survival following hepatectomy for hepatocellular carcinoma: a systematic review and meta-analysis. J Hepatobiliary Pancreat Sci. (2014) 21:639–47. doi: 10.1002/jhbp.124, PMID: 24867654

[8] Forner A Reig ME de Lope CR Bruix J . Current strategy for staging and treatment: the BCLC update and future prospects. Semin Liver Dis. (2010) 30:61–74. doi: 10.1055/s-0030-1247133, PMID: 20175034

[9] Clinical Practice Guidelines EASL . Management of hepatocellular carcinoma. J Hepatol. (2018) 69:182–236. doi: 10.1016/j.jhep.2018.03.019, PMID: 29628281

[10] Xie D Shi J Zhou J Fan J Gao Q . Clinical practice guidelines and real-life practice in hepatocellular carcinoma: A Chinese perspective. Clin Mol Hepatol. (2023) 29:206–16. doi: 10.3350/cmh.2022.0402, PMID: 36545708 PMC10121293

[11] Cescon M Vetrone G Grazi GL Ramacciato G Ercolani G Ravaioli M . Trends in perioperative outcome after hepatic resection: analysis of 1500 consecutive unselected cases over 20 years. Ann Surg. (2009) 249:995–1002. doi: 10.1097/SLA.0b013e3181a63c74, PMID: 19474679

[12] Llovet JM Brú C Bruix J . Prognosis of hepatocellular carcinoma: the BCLC staging classification. Semin Liver Dis. (1999) 19:329–38. doi: 10.1055/s-2007-1007122, PMID: 10518312

[13] Citterio D Facciorusso A Sposito C Rota R Bhoori S Mazzaferro V . Hierarchic interaction of factors associated with liver decompensation after resection for hepatocellular carcinoma. JAMA Surg. (2016) 151:846–53. doi: 10.1001/jamasurg.2016.1121, PMID: 27248425

[14] Han HS Shehta A Ahn S Yoon YS Cho JY Choi Y . Laparoscopic versus open liver resection for hepatocellular carcinoma: Case-matched study with propensity score matching. J Hepatol. (2015) 63:643–50. doi: 10.1016/j.jhep.2015.04.005, PMID: 25872167

[15] Ciria R Gomez-Luque I Ocaña S Cipriani F Halls M Briceño J . A systematic review and meta-analysis comparing the short- and long-term outcomes for laparoscopic and open liver resections for hepatocellular carcinoma: updated results from the European guidelines meeting on laparoscopic liver surgery, Southampton, UK, 2017. Ann Surg Oncol. (2019) 26:252–63. doi: 10.1245/s10434-018-6926-3, PMID: 30390167

[16] Sposito C Battiston C Facciorusso A Mazzola M Muscarà C Scotti M . Propensity score analysis of outcomes following laparoscopic or open liver resection for hepatocellular carcinoma. Br J Surg. (2016) 103:871–80. doi: 10.1002/bjs.10137, PMID: 27029597

[17] Nomi T Hirokawa F Kaibori M Ueno M Tanaka S Hokuto D . Laparoscopic versus open liver resection for hepatocellular carcinoma in elderly patients: a multi-center propensity score-based analysis. Surg Endosc. (2020) 34:658–66. doi: 10.1007/s00464-019-06812-z, PMID: 31093748

[18] Qamar AA Grace ND Groszmann RJ Garcia-Tsao G Bosch J Burroughs AK . Platelet count is not a predictor of the presence or development of gastroesophageal varices in cirrhosis. Hepatology. (2008) 47:153–9. doi: 10.1002/hep.21941, PMID: 18161700

[19] Berzigotti A Seijo S Arena U Abraldes JG Vizzutti F García-Pagán JC . Elastography, spleen size, and platelet count identify portal hypertension in patients with compensated cirrhosis. Gastroenterology. (2013) 144:102–111.e101. doi: 10.1053/j.gastro.2012.10.001, PMID: 23058320

[20] Bogner A Reissfelder C Striebel F Mehrabi A Ghamarnejad O Rahbari M . Intraoperative increase of portal venous pressure is an immediate predictor of posthepatectomy liver failure after major hepatectomy: A prospective study. Ann Surg. (2021) 274:e10–7. doi: 10.1097/sla.0000000000003496, PMID: 31356261

[21] Jiang WT Yang J Xie Y Guo QJ Tian DZ Li JJ . Simultaneous partial splenectomy during liver transplantation for advanced cirrhosis patients combined with severe splenomegaly and hypersplenism. World J Gastroenterol. (2021) 27:654–65. doi: 10.3748/wjg.v27.i7.654, PMID: 33642835 PMC7901050

[22] Schrecker C Waidmann O El Youzouri H Trojan J Schnitzbauer AA Bechstein WO . Low platelet count predicts reduced survival in potentially resectable hepatocellular carcinoma. Curr Oncol. (2022) 29:1475–87. doi: 10.3390/curroncol29030124, PMID: 35323324 PMC8947630

[23] Gralnek IM Garcia-Pagan JC Hernández-Gea V . Challenges in the management of esophagogastric varices and variceal hemorrhage in cirrhosis - A narrative review. Am J Med. (2024) 137:210–7. doi: 10.1016/j.amjmed.2023.12.001, PMID: 38128860

[24] Kawaguchi Y Honda G Endo I Cherqui D Kokudo N . Current technical issues for surgery of primary liver cancer. Liver Cancer. (2016) 6:51–8. doi: 10.1159/000449345, PMID: 27995088 PMC5159717

[25] Reig M Forner A Rimola J Ferrer-Fàbrega J Burrel M Garcia-Criado Á . BCLC strategy for prognosis prediction and treatment recommendation: The 2022 update. J Hepatol. (2022) 76:681–93. doi: 10.1016/j.jhep.2021.11.018, PMID: 34801630 PMC8866082

[26] Llovet JM Fuster J Bruix J . Intention-to-treat analysis of surgical treatment for early hepatocellular carcinoma: resection versus transplantation. Hepatology. (1999) 30:1434–40. doi: 10.1002/hep.510300629, PMID: 10573522

[27] Zhong JH Li H Xiao N Ye XP Ke Y Wang YY . Hepatic resection is safe and effective for patients with hepatocellular carcinoma and portal hypertension. PloS One. (2014) 9:e108755. doi: 10.1371/journal.pone.0108755, PMID: 25268959 PMC4182657

[28] He W Zeng Q Zheng Y Chen M Shen J Qiu J . The role of clinically significant portal hypertension in hepatic resection for hepatocellular carcinoma patients: a propensity score matching analysis. BMC Cancer. (2015) 15:263. doi: 10.1186/s12885-015-1280-3, PMID: 25886495 PMC4399206

[29] Zheng J Feng X Liang Y Cai J Shi Z Kirih MA . Safety and feasibility of laparoscopic liver resection for hepatocellular carcinoma with clinically significant portal hypertension: a propensity score-matched study. Surg Endosc. (2021) 35:3267–78. doi: 10.1007/s00464-020-07763-6, PMID: 32632488

[30] Cucchetti A Ercolani G Vivarelli M Cescon M Ravaioli M Ramacciato G . Is portal hypertension a contraindication to hepatic resection? Ann Surg. (2009) 250:922–8. doi: 10.1097/SLA.0b013e3181b977a5, PMID: 19855258

[31] Aliseda D Zozaya G Martí-Cruchaga P Herrero I Iñarrairaegui M Argemí J . The impact of portal hypertension assessment method on the outcomes of hepatocellular carcinoma resection: A meta-analysis of matched cohort and prospective studies. Ann Surg. (2024) 280:46–55. doi: 10.1097/sla.0000000000006185, PMID: 38126757

[32] de Franchis R Bosch J Garcia-Tsao G Reiberger T Ripoll C . Baveno VII - Renewing consensus in portal hypertension. J Hepatol. (2022) 76:959–74. doi: 10.1016/j.jhep.2021.12.022, PMID: 35120736 PMC11090185

[33] Cucchetti A Cescon M Golfieri R Piscaglia F Renzulli M Neri F . Hepatic venous pressure gradient in the preoperative assessment of patients with resectable hepatocellular carcinoma. J Hepatol. (2016) 64:79–86. doi: 10.1016/j.jhep.2015.08.025, PMID: 26325538

[34] Chen ZL Yao LQ Pu JL Wu H Xu XF Chen TH . Impact of concurrent splenectomy and esophagogastric devascularization on surgical outcomes of partial hepatectomy for hepatocellular carcinoma in patients with clinically significant portal hypertension: A multicenter propensity score matching analysis. Eur J Surg Oncol. (2022) 48:1078–86. doi: 10.1016/j.ejso.2021.11.118, PMID: 34838392

[35] Chong CC Fuks D Lee KF Zhao JJ Choi GH Sucandy I . Propensity score-matched analysis comparing robotic and laparoscopic right and extended right hepatectomy. JAMA Surg. (2022) 157:436–44. doi: 10.1001/jamasurg.2022.0161, PMID: 35262660 PMC8908223

[36] Chakraborty E Sarkar D . Emerging therapies for hepatocellular carcinoma (HCC). Cancers (Basel). (2022) 14(11):2798. doi: 10.3390/cancers14112798, PMID: 35681776 PMC9179883

[37] Allaire M Thabut D . Portal hypertension and variceal bleeding in patients with liver cancer: Evidence gaps for prevention and management. Hepatology. (2024) 79:213–23. doi: 10.1097/hep.0000000000000291, PMID: 36631021

[38] Sanahuja JM Reverter E Ruiz Á Saenz D Martínez-Ocón J Vidal J . Portal hypertension has no role in perioperative bleeding during liver transplantation with systematic porto-caval shunt. HPB (Oxford). (2023) 25:454–62. doi: 10.1016/j.hpb.2023.01.009, PMID: 36759304

[39] Zong GQ Fei Y Chen J Liu RM . Selective double disconnection for cirrhotic portal hypertension. J Surg Res. (2014) 192:383–9. doi: 10.1016/j.jss.2014.05.065, PMID: 24972739

[40] Singal AG Llovet JM Yarchoan M Mehta N Heimbach JK Dawson LA . AASLD Practice Guidance on prevention, diagnosis, and treatment of hepatocellular carcinoma. Hepatology. (2023) 78:1922–65. doi: 10.1097/hep.0000000000000466, PMID: 37199193 PMC10663390

[41] Liu R Abu Hilal M Wakabayashi G Han HS Palanivelu C Boggi U . International experts consensus guidelines on robotic liver resection in 2023. World J Gastroenterol. (2023) 29:4815–30. doi: 10.3748/wjg.v29.i32.4815, PMID: 37701136 PMC10494765

[42] Guo ZY Hong Y Tu B Cheng Y Wang XM . Laparoscopic liver resection for hepatocellular carcinoma complicated with significant portal hypertension: A propensity score-matched survival analysis. Hepatobiliary Pancreat Dis Int. (2023) 22:358–65. doi: 10.1016/j.hbpd.2022.03.012, PMID: 35370090

[43] Yang W Yan K Goldberg SN Ahmed M Lee JC Wu W . Ten-year survival of hepatocellular carcinoma patients undergoing radiofrequency ablation as a first-line treatment. World J Gastroenterol. (2016) 22:2993–3005. doi: 10.3748/wjg.v22.i10.2993, PMID: 26973395 PMC4779922

[44] Ario K Mizuta T Eguchi Y Kawaguchi Y Oza N Akiyama T . Presence of esophageal varices is a risk factor for non-hemorrhagic death of hepatocellular carcinoma patients treated with radiofrequency ablation. Hepatogastroenterology. (2010) 57:501–6., PMID: 20698217

[45] Kim R Jeong WK Kang TW Song KD Lee MW Ahn SH . Intrahepatic distant recurrence after radiofrequency ablation of hepatocellular carcinoma: relationship with portal hypertension. Acta Radiol. (2019) 60:1609–18. doi: 10.1177/0284185119842830, PMID: 31042068

[46] Xu XF Xing H Han J Li ZL Lau WY Zhou YH . Risk factors, patterns, and outcomes of late recurrence after liver resection for hepatocellular carcinoma: A multicenter study from China. JAMA Surg. (2019) 154:209–17. doi: 10.1001/jamasurg.2018.4334, PMID: 30422241 PMC6439634

[47] Jang CW Kwon HJ Kong H Ha H Han YS Chun JM . Impact of clinically significant portal hypertension on surgical outcomes for hepatocellular carcinoma in patients with compensated liver cirrhosis: a propensity score matching analysis. Ann Hepatobiliary Pancreat Surg. (2016) 20:159–66. doi: 10.14701/ahbps.2016.20.4.159, PMID: 28261694 PMC5325151

[48] Cheng CH Lai Y Hung HC Lee JC Wang YC Wu TH . Recurrence patterns after hepatectomy with very narrow resection margins for hepatocellular carcinoma. Front Surg. (2022) 9:926728. doi: 10.3389/fsurg.2022.926728, PMID: 35910466 PMC9330627

[49] Ortega-Ribera M Gibert-Ramos A Abad-Jordà L Magaz M Téllez L Paule L . Increased sinusoidal pressure impairs liver endothelial mechanosensing, uncovering novel biomarkers of portal hypertension. JHEP Rep. (2023) 5:100722. doi: 10.1016/j.jhepr.2023.100722, PMID: 37151732 PMC10154975

[50] Poisson J Lemoinne S Boulanger C Durand F Moreau R Valla D . Liver sinusoidal endothelial cells: Physiology and role in liver diseases. J Hepatol. (2017) 66:212–27. doi: 10.1016/j.jhep.2016.07.009, PMID: 27423426

[51] Berzigotti A Reig M Abraldes JG Bosch J Bruix J . Portal hypertension and the outcome of surgery for hepatocellular carcinoma in compensated cirrhosis: a systematic review and meta-analysis. Hepatology. (2015) 61:526–36. doi: 10.1002/hep.27431, PMID: 25212123

[52] Kong J Shen S Wang W . Synchronous hepatectomy and splenectomy vs hepatectomy for selected patients with hepatocellular carcinoma and clinically significant portal hypertension: A systematic review and meta-analysis. J Surg Oncol. (2019) 119:964–73. doi: 10.1002/jso.25392, PMID: 30775785

[53] Zhang Q Li Q Shang F Li G Wang M . The benefits of radical treatments with synchronous splenectomy for patients with hepatocellular carcinoma and portal hypertension. Cancers (Basel). (2022) 14(13):3155. doi: 10.3390/cancers14133155, PMID: 35804927 PMC9264870

[54] Zhou C Huang Y Shu C Zhou J Hu X Wang J . Splenectomy before hepatectomy for patients with hepatocellular carcinoma and hypersplenism: A retrospective study. Med (Baltimore). (2021) 100:e24326. doi: 10.1097/md.0000000000024326, PMID: 33530224 PMC7850697

[55] Junrungsee S Vipudhamorn W Lapisatepun W Thepbunchonchai A Chotirosniramit A Lapisatepun W . Portal flow modulation by splenic artery ligation to prevent posthepatectomy liver failure: A randomized controlled trial. Surgery. (2025) 185:109351. doi: 10.1016/j.surg.2025.109351, PMID: 40204604

[56] Li T Wang LL Li YP Gan J Wei XS Mao XR . Predictors of portal vein thrombosis after splenectomy in patients with cirrhosis. World J Hepatol. (2024) 16:241–50. doi: 10.4254/wjh.v16.i2.241, PMID: 38495270 PMC10941749

[57] Martinez-Moreno B Martínez Martínez J Herrera I Guilabert L Rodríguez-Soler M Bellot P . Correlation of endoscopic ultrasound-guided portal pressure gradient measurements with hepatic venous pressure gradient: a prospective study. Endoscopy. (2025) 57:62–7. doi: 10.1055/a-2369-0759, PMID: 39025130

[58] Zhang W Peng C Zhang S Huang S Shen S Xu G . EUS-guided portal pressure gradient measurement in patients with acute or subacute portal hypertension. Gastrointest Endosc. (2021) 93:565–72. doi: 10.1016/j.gie.2020.06.065, PMID: 32615178

[59] Clavien P Barkun J Oliveira MDD Vauthey JN Dindo D Schulick RD . The clavien-dindo classification of surgical complications: five-year experience. Ann Surg. (2009) 250:187–96. doi: 10.1097/SLA.0b013e3181b13ca2, PMID: 19638912

